# Supplementary material for: Investigating the Fibrillar Ultrastructure and Mechanics in Keloid Scars Using In Situ Synchrotron X-ray Nanomechanical Imaging
Source: Materials (Basel). 2022 Mar 1;15(5):1836. doi: 10.3390/ma15051836 (PMC8911729; doi:10.3390/ma15051836)
Supplement: Supplementary file 1 [file materials-15-01836-s001.zip › materials-1354575-supplementary.pdf]

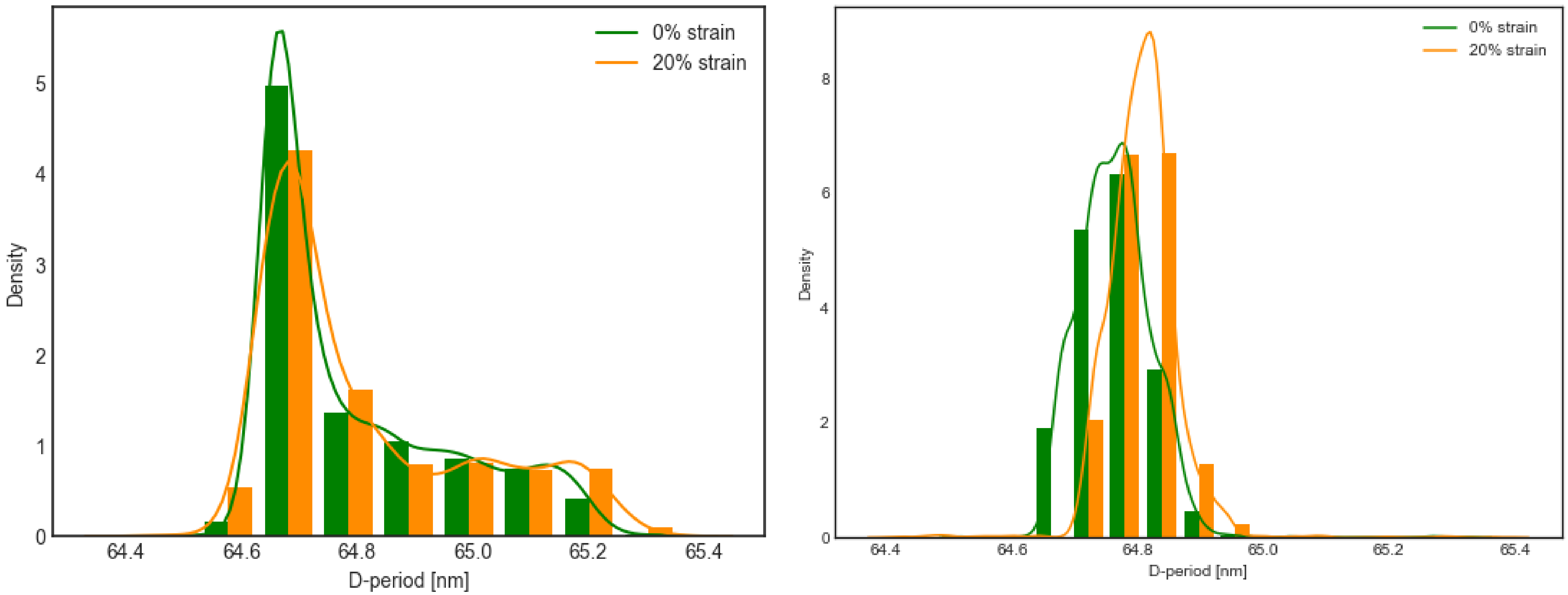

(A) (B)

Supplementary Figure S1: Load-induced changes in D-period distribution for 2 keloid samples, comparing 0% (green) and 20% (orange) tissue strain. Both samples show a rightward shift on application of strain (and load) but due to the inhomogeneous tissue structure the change is not uniform across D-period ranges and within the tissue.
